# Oculocutaneous albinism

**DOI:** 10.1186/1750-1172-2-43

**Published:** 2007-11-02

**Authors:** Karen Grønskov, Jakob Ek, Karen Brondum-Nielsen

**Affiliations:** 1 Kennedy Center. National Research Center for Genetics, visual Impairment and Mental Retardation, Gl. Landevej 7, 2600 Glostrup, Denmark

## Abstract

Oculocutaneous albinism (OCA) is a group of inherited disorders of melanin biosynthesis characterized by a generalized reduction in pigmentation of hair, skin and eyes. The prevalence of all forms of albinism varies considerably worldwide and has been estimated at approximately 1/17,000, suggesting that about 1 in 70 people carry a gene for OCA. The clinical spectrum of OCA ranges, with OCA1A being the most severe type with a complete lack of melanin production throughout life, while the milder forms OCA1B, OCA2, OCA3 and OCA4 show some pigment accumulation over time. Clinical manifestations include various degrees of congenital nystagmus, iris hypopigmentation and translucency, reduced pigmentation of the retinal pigment epithelium, foveal hypoplasia, reduced visual acuity usually (20/60 to 20/400) and refractive errors, color vision impairment and prominent photophobia. Misrouting of the optic nerves is a characteristic finding, resulting in strabismus and reduced stereoscopic vision. The degree of skin and hair hypopigmentation varies with the type of OCA. The incidence of skin cancer may be increased. All four types of OCA are inherited as autosomal recessive disorders. At least four genes are responsible for the different types of the disease (*TYR*, *OCA2*, *TYRP1 *and *MATP*). Diagnosis is based on clinical findings of hypopigmentation of the skin and hair, in addition to the characteristic ocular symptoms. Due to the clinical overlap between the OCA forms, molecular diagnosis is necessary to establish the gene defect and OCA subtype. Molecular genetic testing of *TYR *and *OCA2 *is available on a clinical basis, while, at present, analysis of *TYRP1 *and *MATP *is on research basis only. Differential diagnosis includes ocular albinism, Hermansky-Pudlak syndrome, Chediak-Higashi syndrome, Griscelli syndrome, and Waardenburg syndrome type II. Carrier detection and prenatal diagnosis are possible when the disease causing mutations have been identified in the family. Glasses (possibly bifocals) and dark glasses or photocromic lenses may offer sufficient help for reduced visual activity and photophobia. Correction of strabismus and nystagmus is necessary and sunscreens are recommended. Regular skin checks for early detection of skin cancer should be offered. Persons with OCA have normal lifespan, development, intelligence and fertility.

## Disease name

Oculocutaneous albinism

## Definition

Oculocutaneous albinism (OCA) is a group of four autosomal recessive disorders caused by either a complete lack or a reduction of melanin biosynthesis in the melanocytes resulting in hypopigmentation of the hair, skin and eyes. Reduction of melanin in the eyes results in reduced visual acuity caused by foveal hypoplasia and misrouting of the optic nerve fibres. The clinical spectrum of OCA varies, with OCA1A being the most severe type characterized by a complete lack of melanin production throughout life, while the milder forms OCA1B, OCA2, OCA3 and OCA4 show some pigment accumulation over time. The different types of OCA are caused by mutations in different genes but the clinical phenotype is not always distinguishable, making molecular diagnosis a useful tool and essential for genetic counseling.

## Epidemiology

Albinism can affect people of all ethnic backgrounds and has been extensively studied. Approximately one in 17,000 people have one of the types of albinism [1]. This suggests that about 1 in 70 people carry a gene for OCA. Prevalence of the different forms of albinism varies considerably worldwide, partly explained by the different founder mutations in different genes and the fact that it can be difficult clinically to distinguish between the different subtypes of albinism among the large normal spectrum of pigmentation. OCA2 is the most prevalent form worldwide [2] (Table 1).

**Table 1:** The four known types of OCA

***Gene***	***Gene product***	**Chr. localization**	**Size**	**Disease name**	**Prevalence**
***TYR***	Tyrosinase (TYR)	11q14.3	65 kb (529aa)	OCA1	1:40,000
				OCA1A	
				OCA1B *(Yellow alb.)*	
***OCA2*** ***(p gene)***	OCA2	15q11.2-q12	345 kb (838aa)	OCA2 *(Brown OCA in Africans)*	1:36,000 (white Europeans) 1:3,900–10.000 (Africans)
***TYRP1***	Tyrosinase-related protein 1 (TYRP1)	9p23	17 kb (536aa)	OCA3 (*Rufous OCA*)	Rare (white Europeans, Asians) 1:8,500 (Africans)
***MATP***	Membrane-associated transporter protein (MATP)	5p13.3	40 kb (530aa)	OCA4	Rare (white Europeans) 1:85,000 (Japanese)

• OCA1 has a prevalence of approximately 1 per 40,000 [3] in most populations but is very uncommon among African-Americans.

• In contrast, OCA2 is the most common type of albinism in African Black OCA patients. The overall prevalence of OCA2 is estimated to be 1:36,000 in the USA, but is about 1:10,000 among African Americans [4]. It affects 1 in 3,900 of the population in some parts of the southern part of Africa [5].

• OCA3 or Rufous oculocutaneous albinism has been reported to affect 1:8,500 individuals in Africa, whereas it is very rare in Caucasians and Asiatic populations [6].

• Recently, mutations in a fourth gene were shown to be the cause of albinism, OCA4, [7] and were reported to explain the disease in approximately 5–8% of German patients with albinism [8] but 18% of Japanese patients [9].

## Clinical description

**Figure 1:**
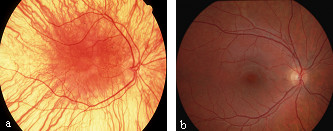
Fundus picture of a patient with albinism (a) and fundus picture of a normal eye (b).

All types of OCA and ocular albinism (OA) have similar ocular findings, including various degrees of congenital nystagmus, hypopigmentation of iris leading to iris translucency, reduced pigmentation of the retinal pigment epithelium, foveal hypoplasia, reduced visual acuity usually in the range 20/60 to 20/400 and refractive errors, and sometimes a degree of color vision impairment [1,10] (Figure 1). Photophobia may be prominent. Iris translucency is demonstrable by slit lamp examination. A characteristic finding is misrouting of the optic nerves, consisting in an excessive crossing of the fibres in the optic chiasma, which can result in strabismus and reduced stereoscopic vision [11]. The abnormal crossing of fibres can be demonstrated by monocular visual evoked potential [12]. Absence of misrouting excludes the diagnosis of albinism.

The degree of skin and hair hypopigmentation varies with the type of albinism but is in general reduced [10] (Table 1).

**Figure 2:**
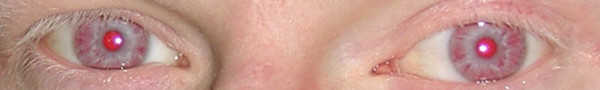
Eyes from a patient with OCA1A. Note that the irises are almost pink, and fully translucent.

• In OCA1A the hair, eyelashes and eyebrows are white, and the skin is white and does not tan. Irises are light blue to almost pink, and fully translucent (Figure 2). Pigment does not develop and amelanotic nevi may be present. The symptoms do not vary with age or race. Visual acuity is 1/10 or less, and photophobia is intense.

• In OCA1B, the hair and skin may develop some pigment with time (after 1 to 3 years), and blue irises may change to green/brown. Temperature-sensitive variants manifest as having depigmented body hairs, and pigmented hairs on hands and feet due to lower temperatures. Visual acuity is 2/10. This phenotype was previously known as yellow albinism.

• In OCA2, the amount of cutaneous pigment may vary, and newborn nearly always have pigmented hair. Nevi and ephelids are common. Iris color varies and the pink eyes seen in OCA1A are usually absent. Visual acuity is usually better than in OCA1, and can reach 3/10. In Africans, brown OCA is associated with light brown hair and skin, and gray irises. Visual acuity may reach 3/10.

• OCA3 results in Rufous or red OCA in African individuals, who have red hair and reddish brown skin (xanthism). Visual anomalies are not always detectable, maybe because the hypopigmentation is not sufficient to alter the development.

• OCA4 cannot be distinguished from OCA2 on clinical findings.

## Etiology

**Figure 3:**
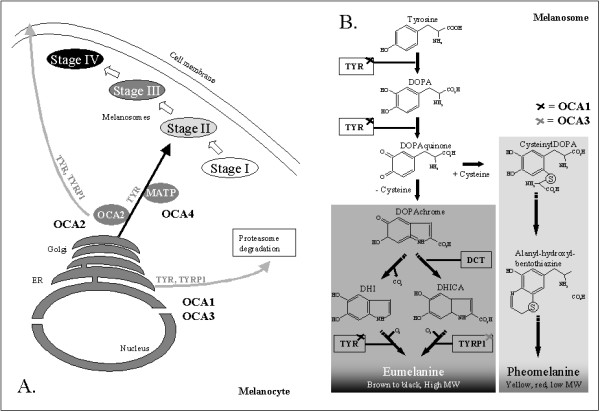
Tyrosinase (TYR) and Tyrosinase-related protein 1 (TYRP1) processing and the melanin biosynthetic pathway in the melanocyte and in the melanosome, respectively. **A**) Schematic representation of melanosome biogenesis in the melanocyte and trafficking of TYR and TYRP1 from the endoplasmatic reticulum (ER) via Golgi apparatus to the developing melanosome. Places are indicated where the transport or sorting of TYR and TYRP1 from the synthesis in the ER to the melanosomes is abolished caused by mutations in the four genes found to be responsible for OCA (OCA1 to OCA4, respectively) (adapted from [49]). **B**) Illustration of the melanin (eumelanin/pheomelanin) biosynthesis pathway in the melanosome. DHI: 5,6-Dihydroxyindole, DHICA: 5,6-Dihydroxyindole-2-carboxylic acid, TYR: tyrosinase, TYRP1: Tyrosinase-related protein 1 (DHICA oxidase), DCT: Dopachrome tautomerase.

OCA is a group of congenital heterogeneous disorders of melanin biosynthesis in the melanocytes (Figure 3). At least four genes are responsible for the different types of OCA (OCA1-4) (Table 1). Most patients are compound heterozygotes, *i.e*. harbouring two different mutations in one of the genes.

• OCA1 (MIM 203100) is caused by mutations in the tyrosinase gene (*TYR*, MIM 606933) on chromosome 11q14.3 [13]. The gene consists of 5 exons spanning about 65 kb of genomic DNA and encoding a protein of 529 amino acids [14]. TYR (EC 1.14.18.1) is a copper-containing enzyme catalysing the first two steps in the melanin biosynthesis pathway, converting tyrosine to L-dihydroxy-phenylalanine (DOPA) and subsequently to DOPAquinone [15]. Mutations completely abolishing tyrosinase activity result in OCA1A, while mutations rendering some enzyme activity result in OCA1B allowing some accumulation of melanin pigment over time. Almost 200 mutations in *TYR *are known [16]. As with all recessive disorders, the "mildest" mutation is determining for the phenotype. It has been shown that mutations in the mouse *Tyr *gene cause the Tyr protein to be retained in the endoplasmic reticulum, with subsequently early degradation [17] (Figure 3).

• Mutations in the *OCA2 *gene (formerly known as the P-gene) (MIM 203200) cause the OCA2 phenotype (MIM 203200) [18]. The gene consists of 24 exons (23 coding), spanning almost 345 kb of genomic DNA in the region 15q11.2-q12, and encoding a protein of 838 amino acids [19]. The OCA2 protein is a 110 kDa integral melanosomal protein with 12 predicted transmembrane domains [18,20]. OCA2 protein is important for normal biogenesis of melanosomes [21,22], and for normal processing and transport of melanosomal proteins such as TYR and TYRP1 [23-26] (Figure 3). TYR stably expressed in a human cell line is retained in perinuclear compartments; this mislocalization can be reverted if OCA2 is co-expressed [27]. It seems that OCA2 exerts at least some of its effects by maintaining an acidic pH in melanosomes [27]. In the Human Gene Mutation Database (HGMD) [16], 72 mutations in *OCA2 *are listed to cause OCA.

• OCA3 (MIM 203290) is caused by mutations in tyrosinase-related protein 1 (*TYRP1*, MIM 115501, 9p23) [28]. *TYRP1 *spans almost 17 kb genomic DNA, and consists of 8 exons encoding a protein of 536 amino acids [29]. TYRP1 is an enzyme in the melanin biosynthesis pathway, catalysing the oxidation of 5,6-dihydroxyindole-2-carboxylic acid (DHICA) monomers into melanin (Figure 3). Studies of mouse melanocytes showed that Tyrp1 functions to stabilize Tyr, and that mutations in *Tyrp1 *cause a delayed maturation and an early degradation of Tyr [17] (Figure 3). Until recently, OCA3 was only known in individuals of African descent, however currently mutations in *TYRP1 *have been identified in both a large Pakistani family [30] and in a Caucasian patient [6].

• Mutations in the membrane-associated transporter protein gene (*MATP*, also known as *SLC45A2*, MIM 606202) cause OCA4 (MIM 606574) [7]. *MATP *consists of 7 exons spanning approximately 40 kb of genomic DNA, mapping to chromosomal position 5p13.3. The MATP protein of 530 amino acids contains 12 putative transmembrane domains and shows sequence and structural similarity to plant sucrose transporters; it is expressed in melanosomal cell lines [31,32]. The function of MATP is still unknown, but studies from Medaka fish show that the MATP protein plays an important role in pigmentation and probably functions as a membrane transporter in melanosomes [31] (Figure 3). Mutations in *MATP *were found for the first time in a Turkish OCA patient [7], and have since been found in German, Japanese and Korean OCA patients [8,9,33,34].

## Diagnostic methods

The diagnosis of OCA is based on clinical findings of hypopigmentation of the skin and hair, in addition to the characteristic ocular symptoms (Figure 1). However, due to the clinical overlap between the OCA subtypes, molecular diagnosis is necessary in order to establish the gene defect and thus the OCA subtype. Molecular genetic testing of *TYR *and *OCA2 *are available on a clinical basis, while at present, analysis of *TYRP1 *and *MATP *is on research basis only. Molecular genetic testing is based on mutational analysis of the genes, by standard screening methods such as denaturing high performance liquid chromatography (DHPLC) or single stranded conformational polymorphism (SSCP), followed by DNA sequencing.

Mutational analysis of *TYR *is complicated by the presence of a pseudogene harbouring sequences highly similar to exon 4 and 5 of *TYR*. This can be overcome either by digestion of pseudogene sequences with restriction enzymes prior to PCR amplification or by use of specific primers only amplifying *TYR *sequences [35].

Due to the presence of numerous polymorphisms, mutational analysis of *OCA2 *is difficult and until a functional assay is available, investigation of control chromosomes in addition to *in silico *analyses of amino acid substitutions are necessary in order to substantiate the probable deleterious effect of a (missense) mutation.

## Genetic counseling and antenatal diagnosis

All four types of OCA are inherited as autosomal recessive disorders. Thus, the parents of an affected child are obligate carriers, the recurrence risk for another affected child is 25%, and healthy sibs are at 67% risk of being carriers. Offspring of an affected person are obligate carriers. Carriers are asymptomatic.

In most cases, there is no previous family history of albinism but the condition does occur in individuals of two generations of a family, so called pseudodominance, and is due to an affected person having children with a person who is a carrier.

In African populations, there is a high frequency of *OCA2 *mutant alleles, hence affected patients in several generations may be seen.

Carrier detection and prenatal diagnosis are possible when the disease causing mutations have been identified in the family. Both disease causing mutations in an affected person have to be identified and established to be on the paternal and maternal chromosome, respectively, before prenatal diagnosis can be performed in pregnancies at 25% risk for an affected child. The testing can be done on DNA extracted from chorion villus sampling (CVS) at 10–12 weeks gestation or on DNA extracted from cultured amniocytes. Preimplantation diagnosis using molecular genetic analysis is also possible in principle, but to our knowledge, this has not been carried out.

Previously, prenatal diagnosis has been performed on skin biopsies from the fetus [36,37]. Requests for prenatal diagnosis for OCA are not common, and may reflect the nature of the condition (not affecting intellectual functions or general health). However, many centers including ours would consider prenatal testing after careful genetic counseling of the parents.

## Management

### Management of eye problems

Reduced visual acuity can be helped in various ways. Clinics specialized in low vision will provide the expertise. Glasses, possibly bifocals, may often be of sufficient help. Photophobia can be helped with dark glasses or photocromic lenses that darken with exposure to bright light. Nystagmus may be helped with contact lenses or surgery of the eye muscles. Certain positions of the head may dampen nystagmus. For strabismus it may be necessary to patch one eye in children to force the non-preferred eye to be used.

Children should be given special attention at school, for instance with high contrast written material, large type textbooks, various optic devices as enlargement machines (closed circuit TV), and the use of computers.

### Skin

Most people with severe forms of OCA do not tan and easily get sunburned. Those forms with a little pigment developing with age may not be very bothered by the sun. Sunscreens are recommended with at least a sun protection factor of 15. Ultraviolet rays can penetrate light T-shirts especially when wet. Now, T-shirts have been developed which protect against the sun even when wet. The incidence of skin cancer is increased in patients with OCA [3]. Since the prevalence of OCA2 is high in Africa, this may pose a serious health problem.

## Differential diagnosis

It has become evident that heterogeneity exists within oculocutaneous albinism, and several disorders with characteristic of OCA in addition to other symptoms have been identified. On the contrary, in Ocular Albinism (OA) the hypopigmentation is limited to the eyes resulting in irides that are blue to brown, nystagmus, strabismus, foveal hypoplasia, abnormal crossing of the optic fibres and reduced visual acuity [38]. The gene *OA1 *is localized on the X chromosome and only fibersboys are affected [39]. In young boys with light complexion, of *e.g*. Scandinavian extraction, some difficulty in the differential diagnosis of OCA versus OA is not uncommon.

Among disorders where albinism is part of a larger syndrome are Hermansky-Pudlak syndrome (HPS), Chediak-Higashi syndrome (CHS), Griscelli Syndrome, and Waardenburg Syndrome type II (WS2). All, except WS2, are inherited as autosomal recessive traits and can be distinguished on the basis of clinical and biochemical criteria. Several subtypes exist within the different diagnoses. Further, an association of hypopigmentation in Prader Willi syndrome and Angelman disease with a deletion on 15q11 has been found, presumable caused by mutations in *OCA2 *[40].

• The Hermansky-Pudlak syndrome is characterized by hypopigmentation and the accumulation of a material called ceroid in tissues throughout the body [41]. Further, patients exhibit severe immunologic deficiency with neutropenia and lack of killer cells [42]. HPS is very rare, except in Puerto Rico where it affects approximately 1 in 1,800 individuals [43]. The most important medical problems in HPS are related to interstitial lung fibrosis, granulomatous colitis and mild bleeding problems due to a deficiency of granules in the platelets [44].

• The Chediak-Higashi syndrome is a rare condition that includes an increased susceptibility to bacterial infections, hypopigmentation, prolonged bleeding time, easy bruisability, and peripheral neuropathy. The skin, hair, and eye pigment is reduced or diluted in CHS [45,46].

• The Griscelli syndrome is a rare disorder with immune impairment or neurological deficit and hypopigmentation of skin and hair, and the presence of large clumps of pigment in hair shafts [47].

• A syndrome of sensory deafness and partial albinism is referred to as the albinism-deafness syndrome or the Waardenburg syndrome [48].

## Prognosis

Lifespan in patients with OCA is not limited, and medical problems are generally not increased compared to those in the general population. As mentioned, skin cancers may occur and regular skin checks should be offered. Development and intelligence are normal. Persons with OCA have normal fertility.

## Unresolved questions

We and others have identified mutations in two alleles in approximately 50% of the patients investigated with genetic screening of the four known OCA genes (*OCA1-4*) (unpublished results). Further, some individuals classified with OCA1 or OCA2 have only one mutation identified. This means that a fraction of patients with albinism still need to be genetically solved. Therefore, more work is needed to establish whether subtle genetic changes in regions not traditionally covered by genetic screening, *i.e*. introns or regulatory domains are the cause of the disease in cases with only one mutation identified. Further, large genomic deletions or single exon deletions not identified by traditional screening methods may explain the disease in a fraction of the patients. In addition, a percentage of genetically unresolved cases might be explained by mutations in not yet identified OCA genes. Finally, the biological function of the gene products of the genes identified as the cause of albinism is not clarified and further elucidation of these mechanisms may give clues to further candidate genes where mutations are the cause of new subtypes of OCA.

## Abbreviations

CHS : Chediak-Higashi syndrome

CVS : Chorion villus biopsy

DOPA : L-dihydroxy-phenylalanine

HPS : Hermansky-Pudlak syndrome

OA : Ocular albinism

OCA : Oculocutaneous albinism

WS2 : Waardenburg Syndrome type II

## Competing interests

The author(s) declare that they have no competing interests.

## Authors' contributions

All authors contributed to a draft of the manuscript and were subsequently involved in revising the manuscript critically for important intellectual content. All authors read and approved the final manuscript.
